# Vascular miR-181b controls tissue factor-dependent thrombogenicity and inflammation in type 2 diabetes

**DOI:** 10.1186/s12933-020-0993-z

**Published:** 2020-02-17

**Authors:** Marco Witkowski, Mario Witkowski, Mona Saffarzadeh, Julian Friebel, Termeh Tabaraie, Loc Ta Bao, Aritra Chakraborty, Andrea Dörner, Bernd Stratmann, Diethelm Tschoepe, Samantha J. Winter, Andreas Krueger, Wolfram Ruf, Ulf Landmesser, Ursula Rauch

**Affiliations:** 1 Charité Centrum 11, Department of Cardiology, Charité – Universitätsmedizin Berlin, Campus Benjamin Franklin, Hindenburgdamm 30, 12200 Berlin, Germany; 2 Research Centre Immunology and Institute of Medical Microbiology and Hygiene, University of Mainz Medical Centre, Mainz, Germany; 3 Center for Thrombosis and Hemostasis, University Medical Center of the Johannes Gutenberg University, Mainz, Germany; 4 Heart and Diabetes Center NRW, Ruhr University of Bochum, Bad Oeynhausen, Germany; 5 Institute for Molecular Medicine, Goethe-University Frankfurt, Frankfurt am Main, Germany

**Keywords:** Diabetes mellitus, Diabetes complications, Thrombosis, Tissue factor, microRNA, Vascular homeostasis, Endothelial cells, Monocytes, PTEN, NFκB

## Abstract

**Background:**

Diabetes mellitus is characterized by chronic vascular inflammation leading to pathological expression of the thrombogenic full length (fl) tissue factor (TF) and its isoform alternatively-spliced (as) TF. Blood-borne TF promotes factor (F) Xa generation resulting in a pro-thrombotic state and cardiovascular complications. MicroRNA (miR)s impact gene expression on the post-transcriptional level and contribute to vascular homeostasis. Their distinct role in the control of the diabetes-related procoagulant state remains poorly understood.

**Methods:**

In a cohort of patients with poorly controlled type 2 diabetes (n = 46) plasma levels of miR-181b were correlated with TF pathway activity and markers for vascular inflammation. In vitro, human microvascular endothelial cells (HMEC)-1 and human monocytes (THP-1) were transfected with miR-181b or anti-miR-181b and exposed to tumor necrosis factor (TNF) α or lipopolysaccharides (LPS). Expression of TF isoforms, vascular adhesion molecule (VCAM) 1 and nuclear factor (NF) κB nuclear translocation was assessed. Moreover, aortas, spleen, plasma, and bone marrow-derived macrophage (BMDM)s of mice carrying a deletion of the first miR-181b locus were analyzed with respect to TF expression and activity.

**Results:**

In patients with type 2 diabetes, plasma miR-181b negatively correlated with the procoagulant state as evidenced by TF protein, TF activity, d-dimer levels as well as markers for vascular inflammation. In HMEC-1, miR-181b abrogated TNFα-induced expression of flTF, asTF, and VCAM1. These results were validated using the anti-miR-181b. Mechanistically, we confirmed a miR-181b-mediated inhibition of importin-α3 (KPNA4) leading to reduced nuclear translocation of the TF transcription factor NFκB. In THP-1, miR-181b reduced both TF isoforms and FXa generation in response to LPS due to targeting phosphatase and tensin homolog (PTEN), a principal inducer for TF in monocytes. Moreover, in miR-181−/− animals, we found that reduced levels of miR-181b were accompanied by increased TF, VCAM1, and KPNA4 expression in aortic tissue as well as increased TF and PTEN expression in spleen. Finally, BMDMs of miR-181−/− mice showed increased TF expression and FXa generation upon stimulation with LPS.

**Conclusions:**

miR-181b epigenetically controls the procoagulant state in diabetes. Reduced miR-181b levels contribute to increased thrombogenicity and may help to identify individuals at particular risk for thrombosis.

## Background

Under diabetic conditions, chronic inflammatory signaling in the vasculature sustains endothelial dysfunction, leukocyte infiltration, and a pro-thrombotic environment [1, 2].

Tissue factor (TF), the membrane-bound receptor for coagulation factor (F) VIIa, is constitutively expressed in the perivascular space but under physiological conditions not present in the vasculature or blood [3]. However, hyperglycemia and pro-inflammatory molecules, such as tumor necrosis factor (TNF)α or lipopolysaccharides (LPS), induce the highly thrombogenic full length (fl) TF and its less thrombogenic isoform alternatively-spliced (as) TF [4–6]. Vessel wall-derived flTF mainly drives inflammatory signaling via protease-activated receptor (PAR)s [7–9], can lead to vascular dysfunction under oxidative stress [10], and may contribute to atherothrombosis through release in TF-positive procoagulant microvesicles (MVs) [11, 12]. In the blood, hematopoietic cells, including monocytes and macrophages, and their released procoagulant MVs represent the main source of TF [13, 14]. When in contact with blood, flTF triggers the FVIIa-dependent generation of FXa prompting thrombin accumulation and clotting [15]. Particularly, patients with poorly controlled diabetes show pathological TF activity in the blood accounting for vascular complications [16].

Recently, a critical role of microRNA (miR)s in the pathogenesis of cardiovascular diseases (CVD) and diabetes-related complications has been demonstrated [17–19]. Binding of specific target transcripts by miRs leads to mRNA degradation and translational repression. We have recently shown that miR-126 and miR-19a target the TF transcript thereby contributing to the epigenetic control of vascular inflammation and coagulation [20, 21]. Thus, a procoagulant state in diabetes can also be considered a result of a defective miR-mediated control of coagulation factors.

Among endothelial miRs, miR-181b emerged as a powerful regulator of vascular homeostasis. Several lines of evidence point to implication of miR-181b into TF-related thrombogenicity: In endothelial cells (ECs) in vitro, miR-181b targets importin-α3 (KPNA4) and thereby reduces nuclear translocation of nuclear factor (NF) κB, one of the main transcription factors for vascular TF expression [22]. Moreover, Henao Mejia et al. found that miR-181b targets phosphatase and tensin homolog (PTEN), a principal inducer for TF expression in hematopoietic cells [23]. In monocytes, PTEN drives LPS-induced TF expression by negatively regulating the phosphoinositide 3-kinase (PI3K)/AKT pathway. Accordingly, PTEN−/− mice exhibit reduced TF activity [24]. In vivo, Lin et al. reported that miR-181b administration reduced arterial thrombosis in a photochemical injury-model [25], a feature that is also observed in mice with reduced vascular TF expression [26, 27]. Finally, miR-181b expression is lowered via hyperglycemia and pro-inflammatory cytokines that are all known to induce TF expression in the vasculature [28].

In the present study, we sought to assess the role of miR-181b in the hemostatic balance in advanced diabetes.

In a cohort of patients with poorly controlled type 2 diabetes, we provide novel evidence that miR-181b expression strongly correlates with reduced activation of the TF-pathway assessed by TF protein, TF activity, and d-dimer levels as well as reduced vascular inflammation. In vitro, TF expression and FXa generation was reduced by miR-181b in human ECs and monocytes. Using animals with a deletion of the first miR-181b locus we provide in vivo evidence that miR-181b contributes to the control of vascular TF activity derived from the endothelium and hematopoietic cells. Our data suggests that the epigenetic control by circulating miR-181b is essential to ensure the hemostatic balance by reducing inflammation-driven vascular TF expression.

## Research design and methods

### Patient study

The study protocol was approved by the local ethics committee and was performed in accordance to the ethics principles in the Declaration of Helsinki. Prior to participation in the study, each patient gave a written informed consent. 46 patients with known diabetes mellitus type 2 admitted for poor glycemic control at the Diabetes Center NRW Bad Oeynhausen, Germany, were included in the study. Table 1 shows the patient characteristics.

**Table 1:** Patient characteristics

Patient characteristics n = 46	Mean ± SD, median [IQR] or percentage	Correlation coefficient vs. miR-181b	*p*-value
Age (years)	65.6 ± 9.3	− 0.053	0.722
Female gender (%)	26.0	0.011	0.941
BMI (kg/m^2^)	30.6 [27.75–35.25]	0.203	0.175
Minimal fasting blood glucose (mg/dL)	93.0 [75.75–128.3]	− 0.105	0.485
Maximal fasting blood glucose (mg/dL)	197.5 [140.5–238.8]	0.146	0.332
Average fasting blood glucose (mg/dL)	139.1 [117.8–166.5]	− 0.012	0.934
HbA1c (%)	8.39 ± 1.61	0.2895	0.051
HbA1c (mmol/mol)	68.1 ± 17.7	0.2895	0.051
CRP (mg/dL)	0.40 [0.14–0.64]	− 0.051	0.733
Leukocytes (N/nL)	7.4 [6.37–9.12]	− 0.295	0.045*
Neutrophils (%)	59.4 [52.03–66.65]	− 0.318	0.031*
Monocytes (%)	6.83 ± 2.29	0.028	0.850
d-Dimers (ng/mL)	377.8 [247.4–523.5]	− 0.343	0.019*
Hemoglobin (g/dL)	14.0 ± 1.2	− 0.089	0.555
Homocysteine (µmol/L)	12.5 [10.95–17.08]	0.071	0.637
thrombocytes (N/nL)	211.9 ± 51.7	− 0.226	0.130
Triglycerides (mg/dL)	186 [110.3–271.0]	0.222	0.136
LDL cholesterol (mg/dL)	113 [88–135]	0.043	0.796
miR-181b-5p	0.169 [0.014–0.470]	1	
hypertension (%)	100		
CAD (%)	41.3	− 0.177	0.236
PAD (%)	26.0	− 0.096	0.521
carotid stenosis (%)	8.6	− 0.133	0.375
history of stroke (%)	10.8	− 0.249	0.094
History of myocardial infarction (%)	15.2	− 0.006	0.964
Medication			
Insulin (%)	69.5	− 0.135	0.370
Metformin (%)	50.0	0.462	< 0.001*
Sulfonylurea (%)	17.3	− 0.146	0.330
Statin (%)	50.0	− 0.040	0.787
Diuretics (%)	60.8	0.164	0.274
ACE inhibitor (%)	47.8	0.114	0.447
Angiotensin receptor blocker (%)	28.2	0.194	0.194
Aldosterone antagonist (%)	8.6	− 0.017	0.908
Calcium antagonist (%)	17.3	− 0.177	0.239
Nitrate vasodilators (%)	13.0	− 0.034	0.822
Acetylsalicylic acid (%)	58.7	− 0.021	0.886
Beta-blocker (%)	50.0	0.194	0.194

N = 46, shown are either Pearson or Spearman correlation coefficients for normally and not normally distributed continuous variables, respectively. For binary variables, a point-biserial correlation was performed. Values presented are mean ± SD, medians [IQR] or percentages

*ACE* angiotensin-converting enzyme, *BMI* body mass index, *CAD* coronary artery disease, *CRP* c-reactive protein, *HbA1c* glycated hemoglobin, *LDL* low-density lipoprotein, *MI* myocardial infarction, *PAD* peripheral artery disease, *TF* tissue factor

*p<0.05

### Animals

Tissues and cells from age- and sex-matched 12–16 weeks old miR-181a/b-1−/− mice (B6.Mirc14^tm1.1Ankr^ as already described [29], termed “miR-181−/−” throughout this article) were kindly provided by Andreas Krueger, Institute for Molecular Medicine in Frankfurt, Germany). These mice carry a constitutive deletion of miR-181a1 and miR-181b1 and were housed under specific pathogen free conditions in the central animal facility at the Institute for Molecular Medicine in Frankfurt, Germany.

### ELISA experiments and glucose measurements

The protein plasma concentrations were assessed by using specific ELISA systems (Table 2) according to the manufacturer’s instructions. Fasting blood glucose levels in patients were measured by a standardized laboratory test, while further glucose levels during the day were obtained by a finger-prick test from capillary blood.

**Table 2:** Material

Application	Materials
ELISA	TF ELISA kit (Sekisui), d-dimer ELISA kit (Diagnostica Stago), and TAT ELISA kit (Siemens Healthcare)
Transfection	Negative control mimic (Dharmacon), 200 nM miR-181b mimic (hsa-miR-181b-5p, HMI0270, Sigma), 200 nM anti-miR-181b (anti-hsa-miR-181b-5p, HSTUD0270, Sigma), 200 nM inhibitor control (Dharmacon), siRNA against PTEN (L-003023-00-0005), (Dharmacon) or a control siRNA (Dharmacon)
Taqman PCR	miR-181b-5p (001098), mouse asTF (custom-made), mouse flTF (Mm00438856_m1), human VCAM1 (Hs01003372), mouse VCAM1 (Mm00449197_m1), human PTEN (Hs01628827_s1), mouse PTEN (Mm00477208_m1), mouse KPNA4 (Mm00434725_m1), human GAPDH (Hs99999905_m1), mouse GAPDH (Mm99999915_g1), U6snRNA (001973), and self-designed FAM-tagged TaqMan^®^ gene expression assays for human flTF and asTF (for details see [44])
SYBR Green PCR	Human GAPDH (forward 5′-AGCCACATCGCTCAGACAC-3′, reverse 5′-GCCCAATACGACCAAATCC-3′), human KPNA4 Forward 5′-CAGGAGATTCTTCCAGCCCTTTGTGT-3′, Reverse 5′-ATTACCATCTGTATTTGTTCATTGCCAGCATC-3′)
Western blot	Human flTF (Sekisui diagnostics, 4501, goat IgG), human asTF (monoclonal rabbit, kindly provided by Prof. Vladimir Bogdanov, University of Cincinnati), human VCAM1 (Cell signaling, 13662S, rabbit IgG), human-KPNA4 (Abcam, ab6039, goat IgG), human PTEN (Cell signaling, 9559, rabbit IgG), human p-NFκB (Cell signaling, 3033, rabbit IgG), human NFκB (Santa Cruz, sc-109, rabbit IgG), human histone H3 (Cell signaling, 9715, rabbit), mouse and human GAPDH (Calbiochem, CB1001, mouse IgG), mouse TF (Sekisui diagnostics, 4515, rabbit IgG), mouse VCAM1 (Cell signaling, 39036, rabbit IgG)
IFA	Mouse TF (Sekisui diagnostics, 4515, rabbit IgG), mouse VCAM1 (Cell signalling, 39036, rabbit IgG), Goat anti Rabbit (Dianova), DAPI (Invitrogen), F-actin probe, Alexa Fluor 647 Phalloidin (Thermo Fisher, A12379)

### TF activity

The measurement of TF activity was performed as described before [2]. The recombinant FVIIa was kindly provided by Novo Nordisk.

### Cell culture

HMEC from ATCC were maintained in MCBD 131 medium (Gibco) supplemented with 10% FBS (Gibco), 100 U/mL penicillin/streptomycin (PAA), 2 mM l-glutamin (PAA), and 0.05 mg/mL hydrocortison. HMEC were used for experiments until the 13th passage. THP-1 cells from ATCC were grown in RPMI 1640 medium (Gibco) supplemented with 10% FBS and 1% penicillin/streptomycin.

### Transfection and stimulation experiments

HMEC-1 and THP-1 were transfected with 200 nM of the oligonucleotides listed in Table 2 using the siRNA transfection reagent interferin (VWR) according to manufacturer’s protocol. 24 h post transfection cells were starved in MCBD 131 medium (Gibco) for 2 h and then stimulated with 10 ng/mL TNFα for 2 h for gene expression analysis and 6 h or 24 h for TF or VCAM1 protein expression, respectively. THP-1 cells were stimulated with LPS (10 µg/mL, Sigma) for 2 h for mRNA expression analysis and 6 h for assessment of TF activity. For siRNA-mediated knock down experiments THP-1 were transfected for 48 h.

### Real time PCR

For real-time PCR, total mRNA was isolated from cultured cells and murine tissue with peqGOLD Trifast (Peqlab) and for patient blood plasma using the mirVana Paris Kit (Life Technologies). Gene expression was determined using GoTaq^®^ Probe qPCR Master Mix (Promega) with FAM-tagged TaqMan^®^ gene expression assays (life Technologies) or via SYBR Green assays using SYBR^®^ Select Master Mix (applied Biosystems) with specific primers (Table 2). Relative gene expression was determined via the comparative C(t) (ΔΔCt) method with GAPDH or 18S ribosomal RNA as endogenous control for mRNA and U6 snRNA for miR.

### Western blot

Western blots were performed as described before [3]. Nuclear and cellular lysates were isolated using the NE-PER™ Nuclear and Cytoplasmic Extraction Reagents (Thermo Fisher Scientific) following the manufacturer’s instructions. Antibodies used are listed in Table 2. The western blots have been performed ≥ 3 times.

### Immunofluorescence

For protein staining aortas and spleen were embedded in Tissue-Tek and immunofluorescence was performed with specific antibodies and probes (Table 2).

### Whole blood microvesicle-associated FXa generation

TF activity of whole blood mouse MVs was assessed as previously described [4]. Briefly, whole blood was drawn by vena cava puncture and 10% citrate added. The cell counts for normalization of the TF activity to the monocyte count was done with a Vetscan automated cell counter. The whole blood was stimulated with 10 µg/mL LPS for 4 h to induce MV release and centrifuged 2 × at 3000*g*. The plasma was centrifuged again for 40 min, 4 °C, at 18000*g* to isolate MVs. MV TF activity was determined by adding 2 nM mouse recombinant FVIIa (kindly provided by H. Ostergaard, Novo Nordisk) and 50 nM FX (Haematologic Technologies) to resuspended MVs in HBS (10 mM Hepes, pH 7.4, 137 mM NaCl, 5.3 mM KCl, 1.5 mM CaCl2). FXa was measured by using the chromogenic substrate Spectrozyme FXa (Sekisui Diagnostics).

### Culture of bone marrow-derived macrophages (BMDMs)

BMDMs were in vitro–differentiated as described before [5]. Briefly, isolated bone marrow cells were cultivated in DMEM supplemented with 20% L cell medium, 10% FCS, 1 mM l-glutamine, penicillin, and streptomycin. On day 7 macrophages were seeded at a density of 1 × 10^6^ per well. On the next day, macrophages were stimulated with 1 µg/mL LPS (Enzo Life Sciences) for 4 h. The cells were then lysed for RNA analysis, western blot or measurement of FXa generation as described above.

### Statistical analysis

The statistical analyses were performed using the software GraphPad Prism 7. Correlation of patient characteristics were performed via Pearson correlation for normally distributed values or Spearman correlation in case of not normal distribution. For binary variables, a point-biserial correlation was performed. Differences between 2 groups were examined using a Mann–Whitney test or student’s t test (2-tailed). For comparisons of 1 parameter between more than two related groups the one-way ANOVA test with Dunn’s multiple comparison as post hoc test was used. For data sets with 4 groups and 2 variables a 2-way ANOVA test with Sidak’s multiple comparison post hoc test was performed. Data are represented as mean ± SEM. p-values < 0.05 are considered statistically significant.

## Results

**Fig. 1:**
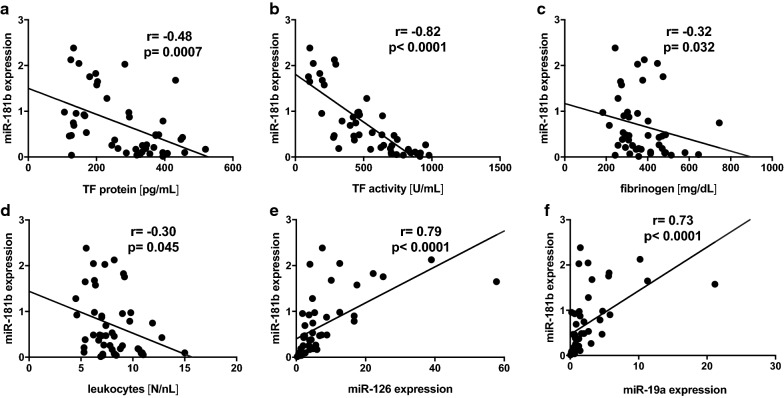
Plasma miR-181b correlates with reduced tissue factor activity and inflammation in diabetes. Plasma of patients with diabetes was analyzed with respect to miR-181b expression. Circulating miR-181b negatively correlated with TF protein (**a**) as well as TF activity (FXa generation) (**b**). Moreover, miR-181b expression was related to lower levels of the pro-inflammatory fibrinogen (**c**) and lower leukocyte counts (**d**). We also observed a correlation of miR-181b with the TF-specific miR-126 (**e**) and miR-19a (**f**). n = 46. Upon normality testing a Spearman or Pearson correlation was performed, r-values and p-values are indicated

We hypothesized an association of miR-181b with vascular TF expression in the setting of diabetes. Therefore, 46 patients with type 2 diabetes admitted to a diabetes center because of insufficient glycemic control (mean HbA1c 8.39 ± 1.61% or 68.1 ± 17.7 mmol/mol) were enrolled in the study. Compared to healthy control subjects with normal TF protein levels in the plasma (median TF protein 102.2 pg/mL [*Q1* 75.8, *Q3* 136.1], Additional file 1: Table S1), the enrolled diabetics presented a pro-thrombotic state (median TF protein 291.3 pg/mL [*Q1 165.3*, *Q3 373.9*] and median TF activity 496.8 U/mL [*Q1 293.4*, *Q3 727.7*]). No association of miR-181b with blood glucose levels was observed. However, circulating miR-181b showed a strong negative correlation with TF protein (Fig. 1a, r = − 0.48, p < 0.001) and TF activity (Fig. 1b, r = − 0.82, p < 0.0001). Moreover, miR-181b correlated with reduced levels of d-dimers   of the TF pathway (r = − 0.34, p < 0.05, Table 1). Furthermore, miR-181b was related to a lower grade of inflammation as evidenced by a negative correlation with fibrinogen and leukocyte count (Fig. 1c, d). In line with this, miR-181b was positively associated with metformin treatment that is known to reduce vascular inflammation (r = 0.462, p < 0.001, Table 1). Plasma miR-181b also correlated with the anti-thrombotic miR-126 and miR-19a (r = 0.79, p < 0.0001 and r = 0.73, p < 0.0001, respectively, Fig. 1e, f).

**Fig. 2:**
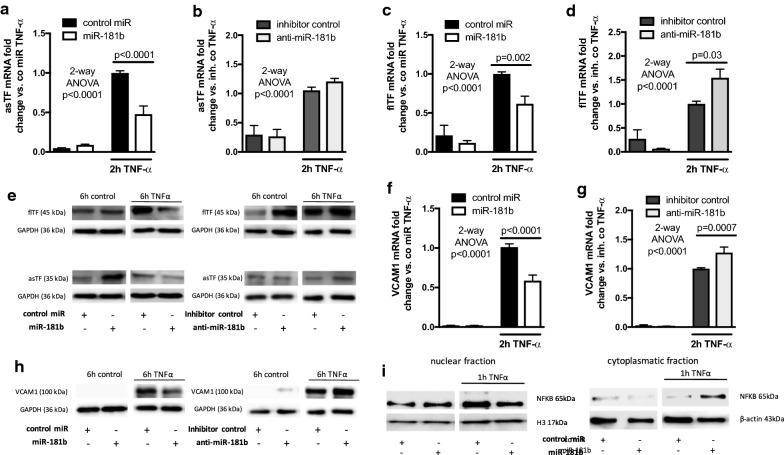
miR-181b reduces the inflammation-induced expression of tissue factor in HMEC-1. HMEC-1 were transfected with a control mimic or miR-181b as well as a negative control inhibitor or anti-miR-181b. The cells were left untreated or stimulated with TNFα for 2 h for mRNA analysis of asTF (**a**, **b**) and flTF (**c**, **d**) or 6 h for TF protein (**e**). VCAM1 mRNA expression (**f**, **g**) and protein (**h**) in HMEC-1 transfected with miR-181b or anti-miR-181b following TNFα for 2 h and 6 h, respectively. Nuclear extracts (**i**, left panel) and cytoplasmatic extracts (**i**, right panel) of HMEC transfected with miR-181b or a control mimic at rest or stimulated with TNFα for 1 h subjected to western blot analysis using antibodies against NFκB, histone H3 (H3), or β-actin. n ≥ 3, groups were compared by 2-way ANOVA test with global p-values for treatment with TNFα and Sidak’s multiple comparison post hoc test

In human microvascular endothelial cells (HMEC)-1, hyperglycemia and the pro-inflammatory cytokine TNFα reduced miR-181b levels, whereas it induced both TF isoforms and the NFκB-dependent vascular cell adhesion molecule (VCAM) 1 (Additional file 2: Figure S1A–H). We analyzed the effect of miR-181b on the inflammation-induced TF expression in ECs. HMEC-1 were therefore transfected with miR-181b or anti-miR-181b as well as the appropriate controls and stimulated with 10 ng/mL TNFα. In miR-181b transfected cells, we observed a reduction of both TF isoforms on the mRNA and protein level in cells stimulated with TNFα for 2 h and 6 h, respectively, whereas anti-miR-181b increased asTF and flTF expression under inflammatory conditions (Fig. 2a–e). We confirmed that miR-181b reduces and anti-miR-181b induces VCAM1 in HMEC-1 (Fig. 2f–h). As the mechanism for inhibition of TNFα-induced TF expression we found a downregulation of KPNA4 in HMEC-1 mRNA and protein (Additional file 3: Figure S2A, B) consistent with a decrease in NFκB nuclear translocation exhibited by miR-181b (Fig. 2i).

**Fig. 3:**
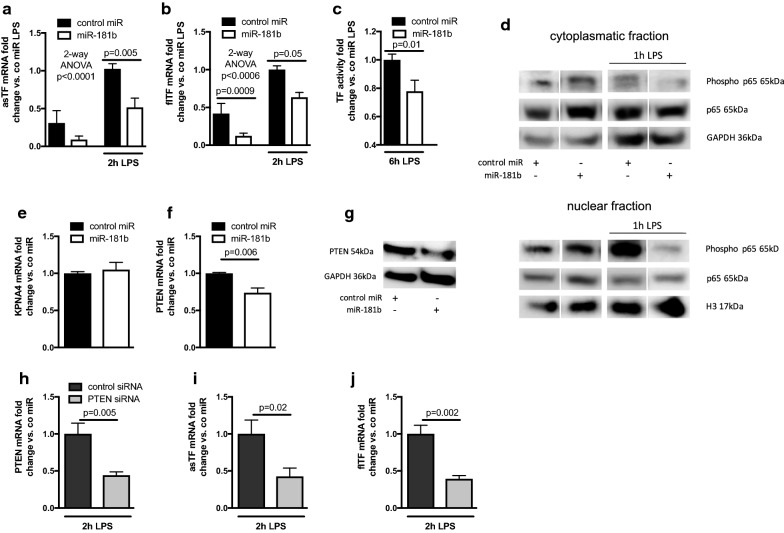
miR-181b inhibits LPS-induced tissue factor activity in THP-1. THP-1 cells were transfected with miR-181b and stimulated with LPS for 2 h to assess asTF and flTF mRNA (**a**, **b**) or 6 h for TF activity (**c**). THP-1 were transfected with a control mimic or miR-181b and then stimulated with LPS for 1 h. Cytoplasmatic extracts (**d**, upper panel) and nuclear extracts (**d**, lower panel) were then subjected to western blot analysis using antibodies against phospho-NFκB (phospho p65), NFκB (p65), histone H3 (H3), or GAPDH (bands show samples with equal loading of the same gel. The original blots can be found in Additional file 3: Figure S3). KPNA4 mRNA (**e**), PTEN mRNA (**f**) and protein (**g**) in THP-1 transfected with a control miR or miR-181b. THP-1 were transfected with a control siRNA or siRNA against PTEN. PTEN knock down was confirmed via quantification of PTEN mRNA (**h**) and led to reduced mRNA levels of flTF (**i**) and asTF (**j**). n ≥ 3, groups were compared by 2-way ANOVA test with global p-values for treatment with LPS and Sidak’s multiple comparison post hoc test (**a**, **b**) or student’s t-test

To assess whether miR-181b also reduces TF expression in human monocytes, the main source of TF activity in the blood, THP-1 cells were transfected with miR-181b and then stimulated with LPS to induce TF expression. Due to the low endogenous expression level of miR-181b in monocytes, a gain of function approach was used. We found that miR-181b reduced mRNA levels of both TF isoforms (Fig. 3a, b) and FXa generation (Fig. 3c) following LPS treatment of THP-1. In contrast to ECs, LPS-induced NFκB was not restricted to the cytoplasmatic compartment in THP-1 (Fig. 3d, upper panel) nor were KPNA4 mRNA levels reduced by miR-181b (Fig. 3e). However, miR-181b led to a marked reduction in NFκB phosphorylation and subsequent nuclear translocation (Fig. 3d, both panels) suggesting an upstream effect on NFκB activation. We suspected that control of PTEN expression may contribute to the miR-181b-mediated reduction of TF activity in monocytes. Indeed, PTEN expression was reduced in THP-1 cells transfected with miR-181b (Fig. 3f, g). siRNA-mediated knock down of PTEN reduced LPS-induced expression of flTF and asTF in THP-1 and phenocopied the miR-181b effect (Fig. 3h–j).

**Fig. 4:**
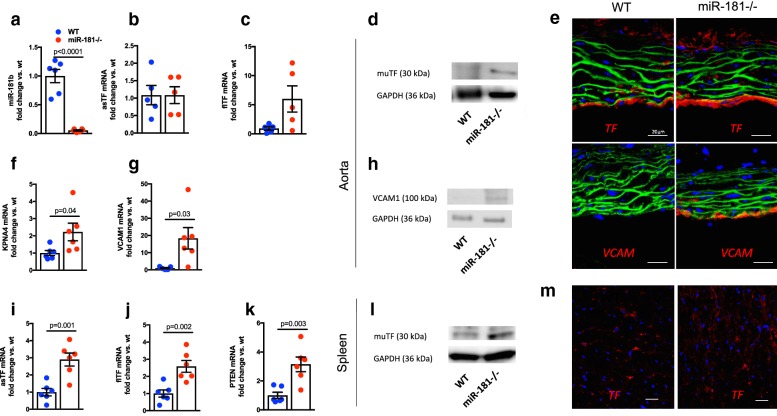
Loss of miR-181b induces tissue factor and VCAM1 expression in vivo. Aortas and spleen of miR-181−/− mice were analyzed with respect to mRNA and protein expression. miR-181b (**a**), asTF mRNA (**b**), flTF mRNA (**c**), murine (mu) TF protein (**d**) and immunofluorescence (IF) images using an anti-mouse (mu) TF antibody (red), DAPI (blue), and a F-actin probe (green) (**e** upper panel) in aortic segments. KPNA4 mRNA (**f**), VCAM1 mRNA (**g**), VCAM1 protein (**h**), and IF images using a mouse VCAM1-specific antibody (red) (**e** lower panel) in aortic tissue. Expression of asTF mRNA (**i**), flTF mRNA (**j**), PTEN mRNA (**k**), muTF protein (**l**), and IF for muTF (red)(M) in spleen of wt and miR-181−/− animals. n = 4–6 animals per group, compared by Mann–Whitney or student’s t-test

To assess the significance of our experimental findings in vivo, animals carrying a deletion of the first miR-181b locus (as well as miR-181a-1; B6.Mirc14^tm1.1Ankr^, here referred to as miR-181−/− mice) with reduced vascular miR-181b expression were analyzed (Fig. 4a). While no change for asTF mRNA was observed, we found an increase in flTF mRNA and protein expression in miR-181−/− aortas as compared to wt mice, (Fig. 4b–d). Immunofluorescence using a murine TF-specific antibody revealed that the rise in TF protein in miR-181−/− aortas originated from the endothelial layer (Fig. 4e, upper panel). Moreover, expression of KPNA4 (Fig. 4f) as well as VCAM1 mRNA and protein (Fig. 4g, h, e lower panel) were increased in aortas of miR-181−/− animals. To assess TF expression in hematopoietic cells, the spleen of miR-181−/− mice was analyzed. Here, miR-181b was reduced (Additional file 5: Figure S4a) while both TF isoforms and PTEN mRNA were increased as compared to wt animals (Fig. 4i–m).

**Fig. 5:**
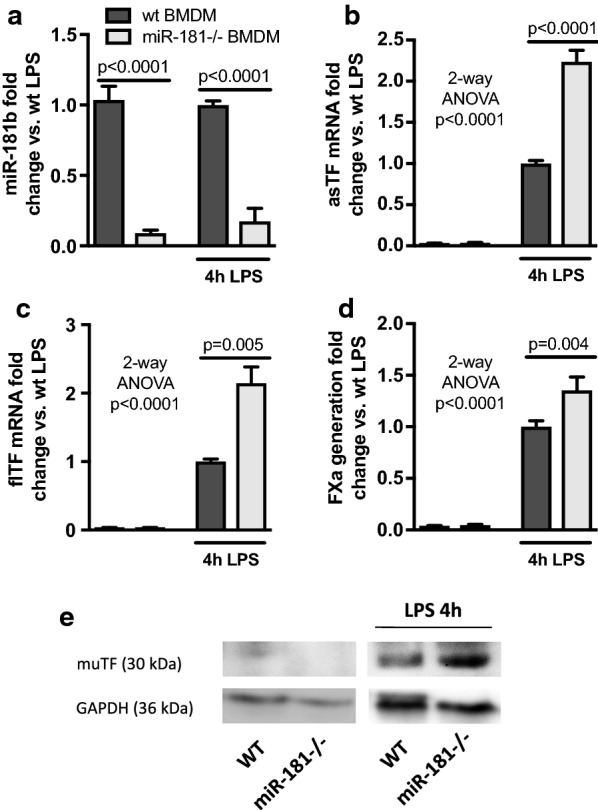
miR-181b deletion in bone marrow-derived macrophages leads to increased TF expression and FXa generation upon inflammation. Bone marrow-derived macrophages (BMDM) were isolated from wt mice or miR-181−/− animals and cultured. The cells were either left untreated or stimulated with 1 µg/mL LPS for 4 h. Next, the miR-181b expression (**a**), asTF mRNA (**b**), flTF mRNA (**c**), FXa generation (**d**), and muTF protein (**e**) were measured. n = 3–6 animals per group, groups were compared by 2-way ANOVA test with global p-values for treatment with LPS and Sidak’s multiple comparison post hoc test

To investigate the blood procoagulant activity of miR-181b−/− animals, whole blood was stimulated with LPS and the FXa generation of MVs assessed. We did not observe a difference in MV-associated FXa generation between blood of wt and ko animals (Additional file 5: Figure S4B). In line, no differences in plasma thrombin anti-thrombin complexes was seen between wt and ko animals (Additional file 5: Figure S4C). However, isolated bone marrow-derived macrophages (BMDM), that have been shown to contribute to blood thrombogenicity, showed a strong reduction in miR-181b expression in the miR-181−/− animals (Fig. 5a) and exhibited higher levels of asTF and flTF mRNA (Fig. 5b, c) as well as more TF protein and FXa generation (Fig. 4d, e) in response to LPS than wt cells.

## Discussion

Here, we report that miR-181b controls thrombogenicity in the vasculature and correlates with reduced circulating TF and vascular inflammation in patients with diabetes. Using human ECs and monocytic cells, we demonstrate that miR-181b reduces the inflammation-induced vascular TF expression and FXa generation. In a murine miR-181 ko system, we provide in vivo evidence that loss of miR-181b promotes endothelial and hematopoietic TF expression. Our work demonstrates that miR-181b is a pivotal regulator of thrombogenicity and contributes to vascular homeostasis in the presence of poorly controlled diabetes.

### Low miR-181b: association with a pro-thrombotic and pro-inflammatory state

In the healthy vasculature, circulating miRs epigenetically control pro-inflammatory transcription factors and their gene products to preserve vascular homeostasis [3, 30–32]. However, in a diabetic environment this protective miR transcriptome is disrupted [33]. In particular, diabetes-related loss of TF-specific miRs, such as miR-126 or miR-223 contributes to vascular inflammation and a pro-thrombotic state [33, 34].

Here we show that miR-181b, a key regulator for endothelial function, beta cell function, peripheral insulin sensitivity, and NFκB signaling [22, 28, 35], participates in the control of vascular TF activity in diabetes. In poorly controlled diabetics, miR-181b was associated with reduced activation of the TF pathway, lower leukocyte count, and fibrinogen, which both constitute independent risk factors for cardiovascular mortality among patients with diabetes [36].

In contrast to markers of inflammation, miR-181b levels did not correlate with blood glucose or HbA1c in our cohort. Although we and Sun et al. [28] found that miR-181b expression in HMEC-1 and human umbilical vein endothelial cells (HUVEC) is reduced by high glucose concentrations, Yang et al. [37] reported activation of the miR-181a/b-specific transcription factor signal transducer and activator of transcription (STAT) 3 in HUVEC treated with sera from diabetics pointing to a more complex regulation of miR-181b expression in humans. This may explain why the miR-181b expression in our healthy controls did not differ from those in poorly controlled diabetes (Additional file 6: Figure S5A). In contrast, miR-126 that has been shown to be reduced in diabetes in the population-based Bruneck cohort, was significantly lower in our diabetes cohort compared to healthy controls confirming the quality of our measurements (Additional file 6: Figure S5B). We assume that diabetic conditions beyond glycemia impact miR-181b expression resulting in differential expression levels among diabetics. Although we cannot rule out an impact of co morbidities and anti-diabetic drugs on miR-181b expression in our cohort, it seems likely that chronic inflammation in an advanced state of the disease is a more relevant factor than blood glucose for miR-181b suppression. In accordance, we found co expression of miR-181b with miR-126 and miR-19a that are both reduced by inflammatory cytokines [20, 21].

### TF in the endothelium is reduced by miR-181b under inflammatory conditions

Our data indicates that miR-181b restrains NFκB-depending TF expression in human and murine ECs via targeting of KPNA4. The direct contribution of vessel wall-derived TF to thrombosis has been a matter of debate [4]. Yet, through allosteric activation of FVIIa and/or FXa generation, TF promotes pro-inflammatory PAR signaling leading to sustained vascular dysfunction. Consequently, an EC-specific deletion of TF reduced circulating inflammatory cytokines [38]. Recently, clinical trials provided strong evidence that the TF/VIIa/Xa pathway contributes to vascular complications and pharmacological blockage of FXa at low doses prevented ischemic CVD endpoints [39]. However, inhibition of vascular TF and PARs are challenged by bleeding issues due to their role in hemostasis and platelet function, respectively [40]. Our study identifies miR-181b as a potent regulator for pathological TF-induced vascular inflammation. Further clinical research needs to assess the therapeutic potential of miR-181b in the setting of diabetes and CVD.

### Endothelial miR-181b governs hematopoietic TF activity via PTEN

In the vasculature, hematopoietic cells represent the main pool of circulating TF and contribute to thrombosis [13, 26]. Here, we show that miR-181b reduces TF activity in human monocytes through control of PTEN.

The strong negative correlation of circulating miR-181b with plasma TF activity in our patient cohort suggests that the mostly endothelial-derived miR-181b impacts monocytic TF production in humans. Recent studies reported that ECs can modulate the phenotype of monocytes via release of endothelial MVs. Njock et al. [41] showed that miR-181b along with other anti-inflammatory miRs is enriched in EC-derived MVs. Treatment of recipient THP-1 with EC-MVs or with miR-181b mimics repressed LPS-induced inflammatory gene expression in monocytes. These data along with our study highlight that endothelial miR-181b exerts anti-thrombotic properties beyond the vasculature by also modulating the thrombotic phenotype of monocytic cells and support the hypothesis that the endothelium at least indirectly participates in the regulation of plasma TF activity.

In contrast to ECs, miR-181b did not alter NFκB nuclear translocation in human monocytes. Different host cell factors that preclude down regulation of KPNA4 may explain this observation. However, we are the first to show that miR-181b controls upstream NFκB activation in monocytes via targeting PTEN. The PI3K/AKT pathway was previously shown to negatively regulate LPS-induced TF transcription in human monocytes through downstream control of NFκB along with the TF-specific transcription factors activator protein (AP)-1 and early growth response (EGR)-1 [42]. Consequently, targeting of the PI3K/AKT negative regulator PTEN by miR-181b reduces LPS-induced TF expression and possibly other inflammatory factors.

### Differences of miR-181b’s effect in the murine system

In the murine system, miR-181b deficiency failed to alter monocyte-derived MV-TF activity but increased TF expression in spleen and BMDM.

In line with previous reports showing that miR-181b does not alter NFκB reporter activity of mouse peripheral blood mononuclear cell (PBMC)s [32], we did not observe changes in MV-associated monocytic TF activity or TAT levels. As suggested by Sun et al., differential expression of importin isoforms in mouse monocytes may be the underlying cause. However, our study shows that miR-181b reduces hematopoietic TF production in murine BMDM. Macrophages not only produce TF-positive thromboinflammatory MVs [14] but also account for TF accumulation in atherosclerotic plaques [43]. Reduced TF expression in macrophages may contribute to the observation that miR-181b overexpression in Apo E−/− mice protects from atherosclerosis [32].

### Limitations

miR expression in patient plasma samples was normalized to U6 which can be altered in some diseases. However, this has not been reported in patients with diabetes and our expression profiles in both cohorts are in accordance with previously published data [34]. Moreover, the miR-181−/− mouse model analyzed here carries a deletion of miR-181b-1 and the adjacent miR-181a-1 as well. Due to transcriptional co-regulation and high sequence similarities both miRs share largely the same functions. Accordingly, some of our findings might also be explained by loss of miR-181a.

## Conclusions

**Fig. 6:**
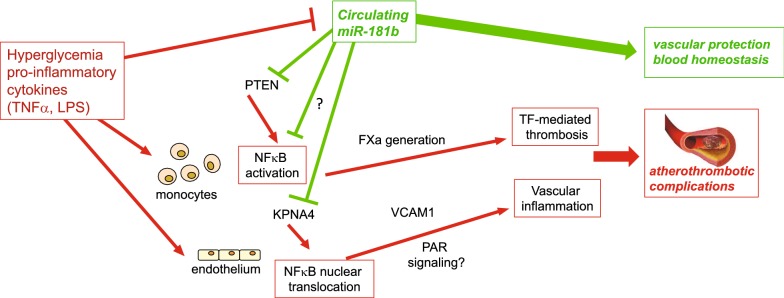
Role of miR-181b in vascular homeostasis

Our work shows that circulating miR-181b contributes to the control of diabetes-related thrombogenicity and vascular inflammation (Fig. 6). Low miR-181b expression may identify patients at particular risk for thromboembolic complications. Vice versa, posttranscriptional control of vascular TF expression by miR-181b represents a potential avenue to improve endothelial dysfunction and thrombosis risk without facing bleeding issues as observed by direct inhibition of inflammatory coagulation factors. Further clinical trials are warranted to explore the prognostic value of miR-181b for prediction of major adverse cardiac events.

## Supplementary information

**Additional file 1: Table S1.** Characteristics of healthy controls. BMI body mass index, HbA1c glycated hemoglobin. Values presented are means ± SD, median [IQR] or percentage.

**Additional file 2: Figure S1.** Vascular inflammation reduces miR-181b expression and induces TF and VCAM1 expression in HMEC-1. HMEC-1 were cultured overnight and exposed to high glucose or stimulated with 10 ng/mL of TNFα. Treatment with 25mM glucose (A) and TNFα (B) caused a reduction of miR-181b expression, while mRNA expression of asTF (C), flTF (D), and VCAM1 (E) was induced after 4h and 6h post stimulation. Western blot analysis showed the protein induction of asTF (F), flTF (G), and VCAM1 (H) following 6 h or 24 h TNFα stimulation. n ≥ 3, p-values by ANOVA test with Dunn’s multiple comparison post hoc test.

**Additional file 3: Figure S2.** miR-181b reduces KPNA4 expression in HMEC-1. KPNA4 mRNA (A) and protein (B) expression in HMEC-1 transfected with a control mimic or miR-181b. n ≥ 3, p-value by student’s t test.

**Additional file 4: Figure S3.** Original western blot showing protein abundance of NFκB, phospho-NFκB, histone 3, and GAPDH in nuclear and cytoplasmic extracts from THP-1 cell transfected with miR-181b or a control mimic under basal conditions or presence of LPS.

**Additional file 5: Figure S4.** No alteration of blood-borne TF activity in miR-181−/− animals. miR-181b expression in spleen tissue of wt and miR-181−/− animals (A). MV-derived FXa generation (B) and TAT complexes (C) in plasma of wt and miR-181−/− animals stimulated with LPS for 4 h. n = 4–5 animals per group, comparison by Mann Whitney or student’s t test.

**Additional file 6: Figure S5.** Healthy controls have higher miR-126 but not miR-181b expression than patients with type 2 diabetes. Expression of miR-181b (A) or miR-126 (B) in heathy controls and the cohort with poorly controlled type 2 diabetes. n = 20 for controls, n = 46 for poorly controlled diabetes, p-values by Mann Whitney test.

## Data Availability

The datasets used during the study are available from the corresponding author on reasonable request.

## Abbreviations

asTF: Alternatively-spliced tissue factor
BMDM: Bone marrow-derived macrophages
flTF: Full-length tissue factor
HMEC: Human microvascular endothelial cells
LPS: Lipopolysaccharide
miR: microRNA
TNF: Tumor necrosis factor
VCAM1: Vascular cell adhesion molecule 1
