# Supplementary material for: Vascular miR-181b controls tissue factor-dependent thrombogenicity and inflammation in type 2 diabetes
Source: Cardiovasc Diabetol. 2020 Feb 17;19:20. doi: 10.1186/s12933-020-0993-z (PMC7027062; doi:10.1186/s12933-020-0993-z)
Supplement: Supplementary file 1 — Additional file 1: Table S1. Characteristics of healthy controls. BMI body mass index, HbA1c glycated hemoglobin. Values presented are means ± SD, median [IQR] or percentage. [file 12933_2020_993_MOESM1_ESM.docx]

**Table S1**

| ***Patient characteristics***  ***n=20*** | ***mean*** ***± SD, median*** *[****IQR****]* ***or percentage*** | ***Correlation coefficient vs. miR-181b*** | ***p-value*** |
| --- | --- | --- | --- |
| age (years) | *48.7 ± 7.3* | *-0.2812* | *0.229* |
| Female gender (%) | *35.0* | *0.106* | *0.655* |
| BMI (kg/m^2^) | *27.0 ± 4.2* | *0.1803* | *0.446* |
| HbA1c (%) | *5.27 ± 0.2* | *0.001* | *0.996* |
| HbA1c (mmol/mol) | *34.0 ± 2.2* | *0.001* | *0.996* |
| Plasma Tissue Factor (pg/mL) | *102.2 [75.8-136.1]* | *0.063* | *0.797* |
