# Supplementary figures and images for: Vascular miR-181b controls tissue factor-dependent thrombogenicity and inflammation in type 2 diabetes
Source: Cardiovasc Diabetol. 2020 Feb 17;19:20. doi: 10.1186/s12933-020-0993-z (PMC7027062; doi:10.1186/s12933-020-0993-z)

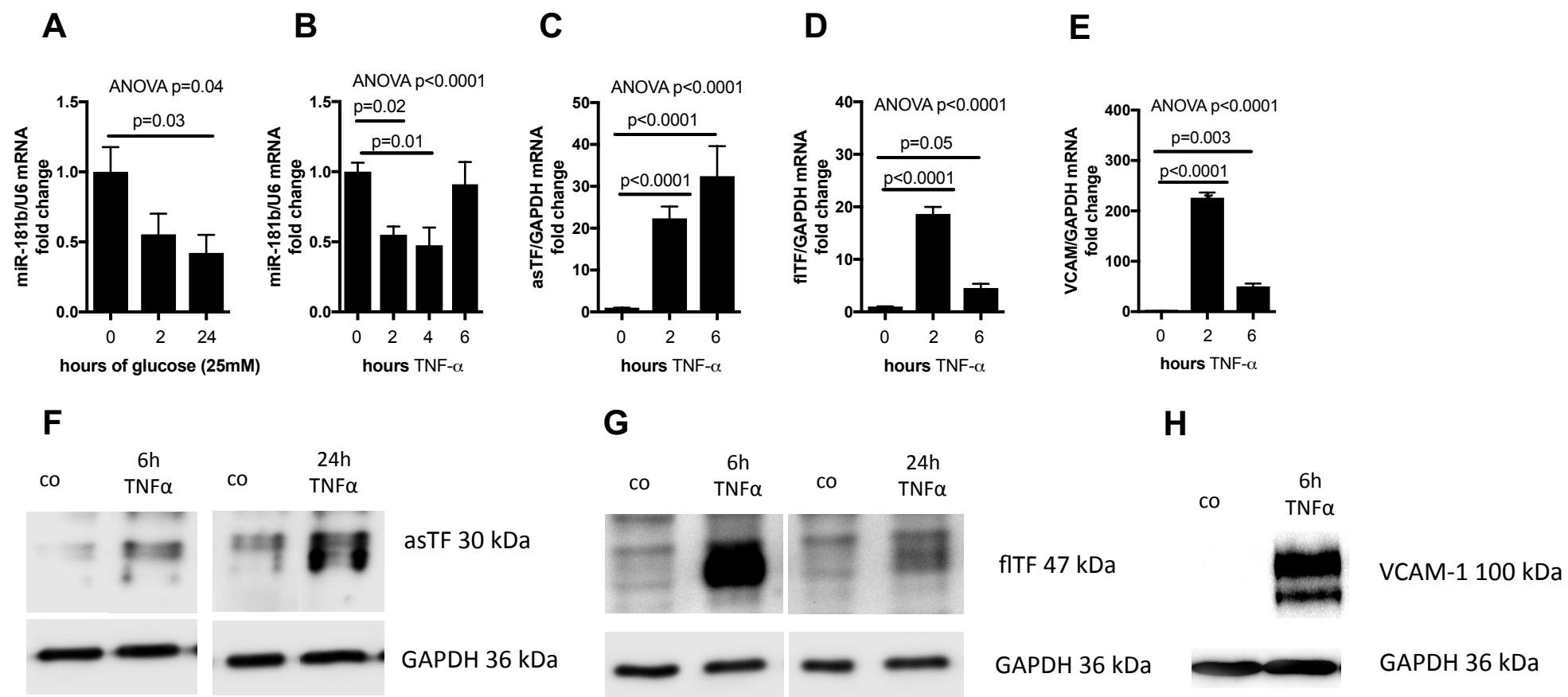

**Figure S1**

Supplement: Supplementary file 2 — Additional file 2: Figure S1. Vascular inflammation reduces miR-181b expression and induces TF and VCAM1 expression in HMEC-1. HMEC-1 were cultured overnight and exposed to high glucose or stimulated with 10 ng/mL of TNFα. Treatment with 25mM glucose (A) and TNFα (B) caused a reduction of miR-181b expression, while mRNA expression of asTF (C), flTF (D), and VCAM1 (E) was induced after 4h and 6h post stimulation. Western blot analysis showed the protein induction of asTF (F), flTF (G), and VCAM1 (H) following 6 h or 24 h TNFα stimulation. n ≥ 3, p-values by ANOVA test with Dunn’s multiple comparison post hoc test. [file 12933_2020_993_MOESM2_ESM.pdf]

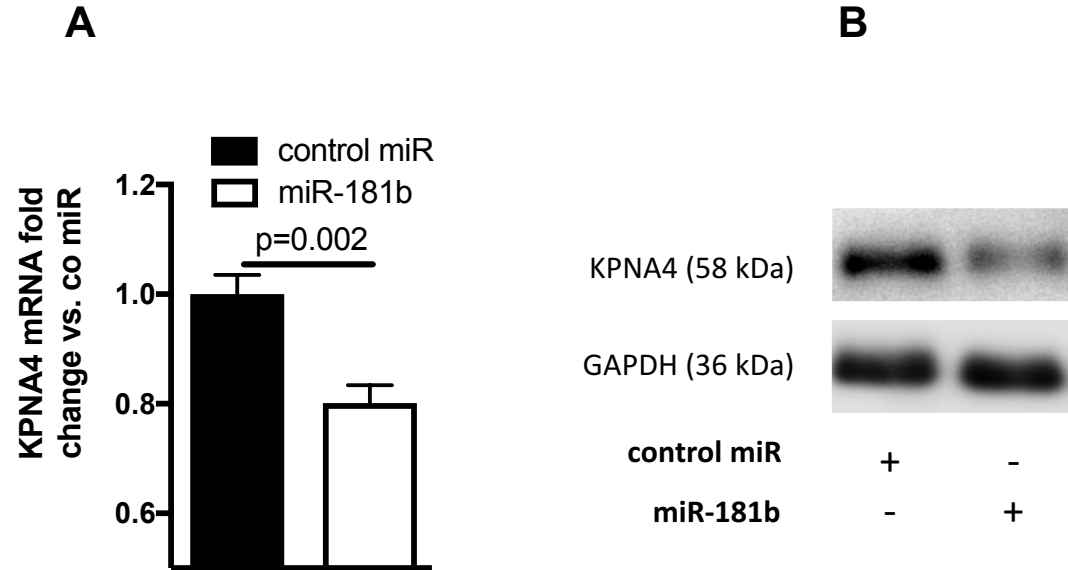

**Figure S2**

Supplement: Supplementary file 3 — Additional file 3: Figure S2. miR-181b reduces KPNA4 expression in HMEC-1. KPNA4 mRNA (A) and protein (B) expression in HMEC-1 transfected with a control mimic or miR-181b. n ≥ 3, p-value by student’s t test. [file 12933_2020_993_MOESM3_ESM.pdf]

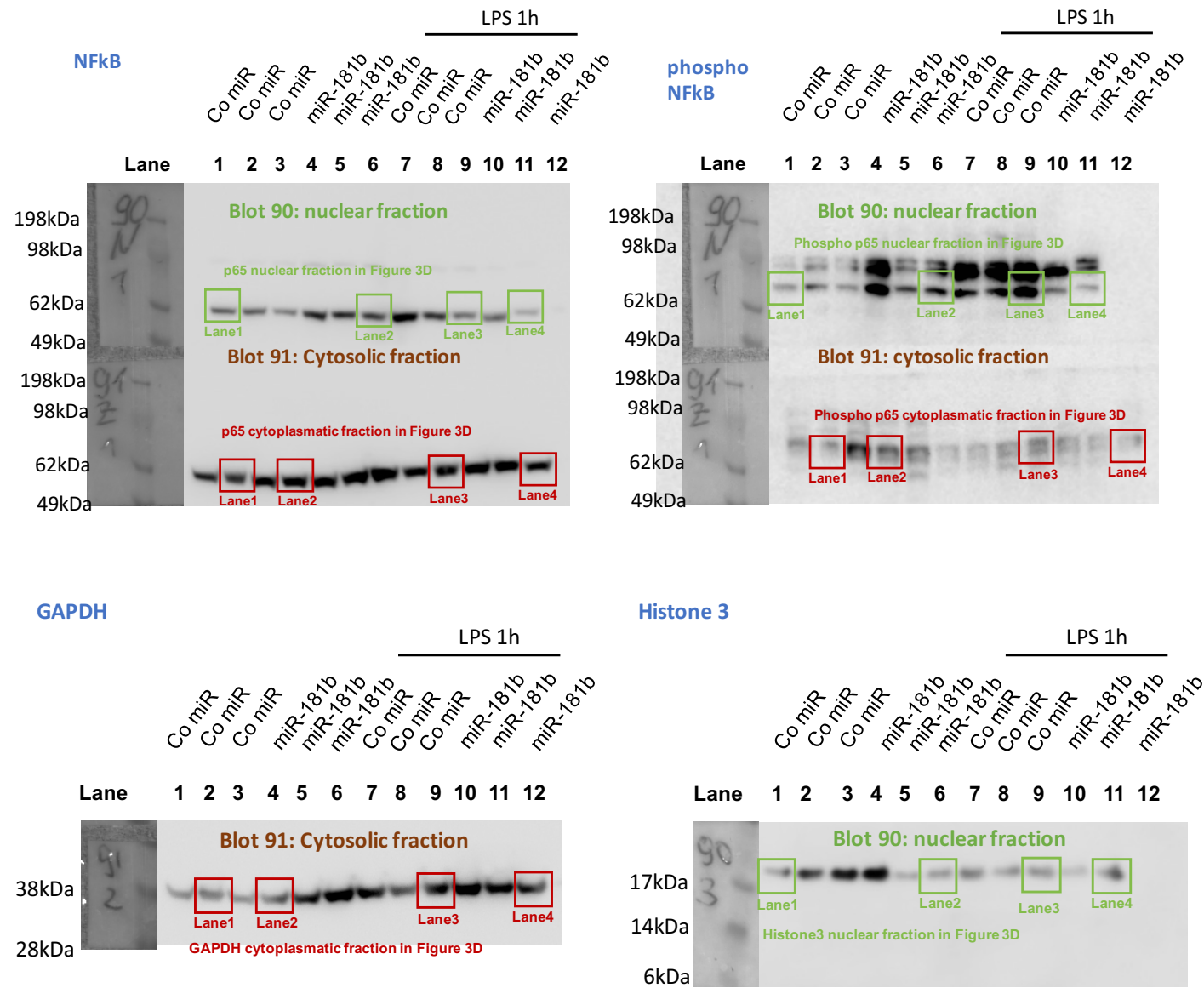

**Figure S3**

Supplement: Supplementary file 4 — Additional file 4: Figure S3. Original western blot showing protein abundance of NFκB, phospho-NFκB, histone 3, and GAPDH in nuclear and cytoplasmic extracts from THP-1 cell transfected with miR-181b or a control mimic under basal conditions or presence of LPS. [file 12933_2020_993_MOESM4_ESM.pdf]

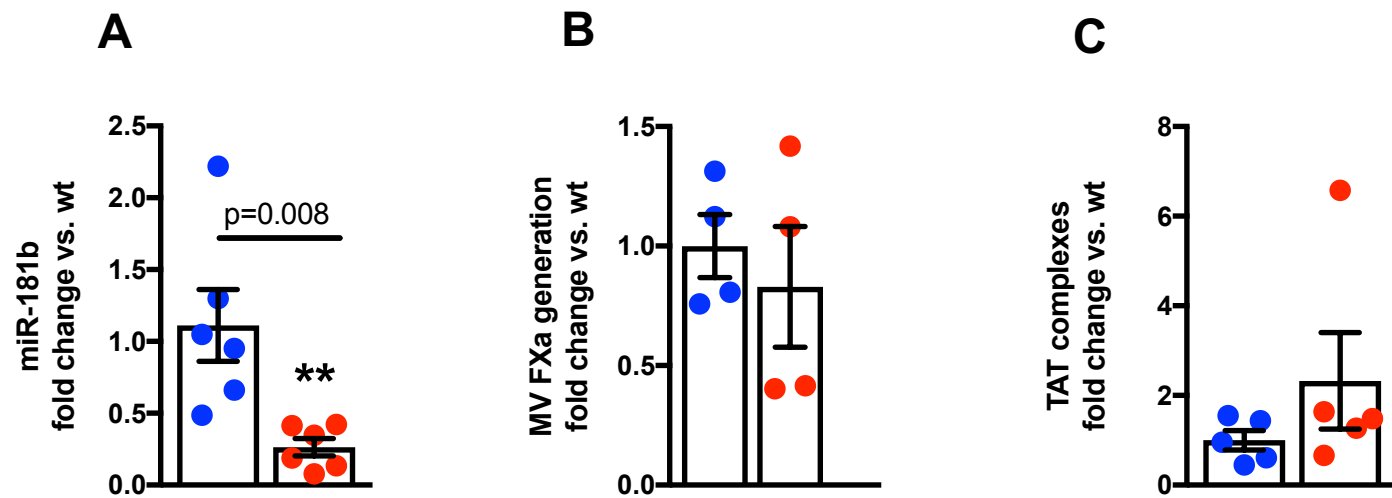

**Figure S4**

Supplement: Supplementary file 5 — Additional file 5: Figure S4. No alteration of blood-borne TF activity in miR-181−/− animals. miR-181b expression in spleen tissue of wt and miR-181−/− animals (A). MV-derived FXa generation (B) and TAT complexes (C) in plasma of wt and miR-181−/− animals stimulated with LPS for 4 h. n = 4–5 animals per group, comparison by Mann Whitney or student’s t test. [file 12933_2020_993_MOESM5_ESM.pdf]

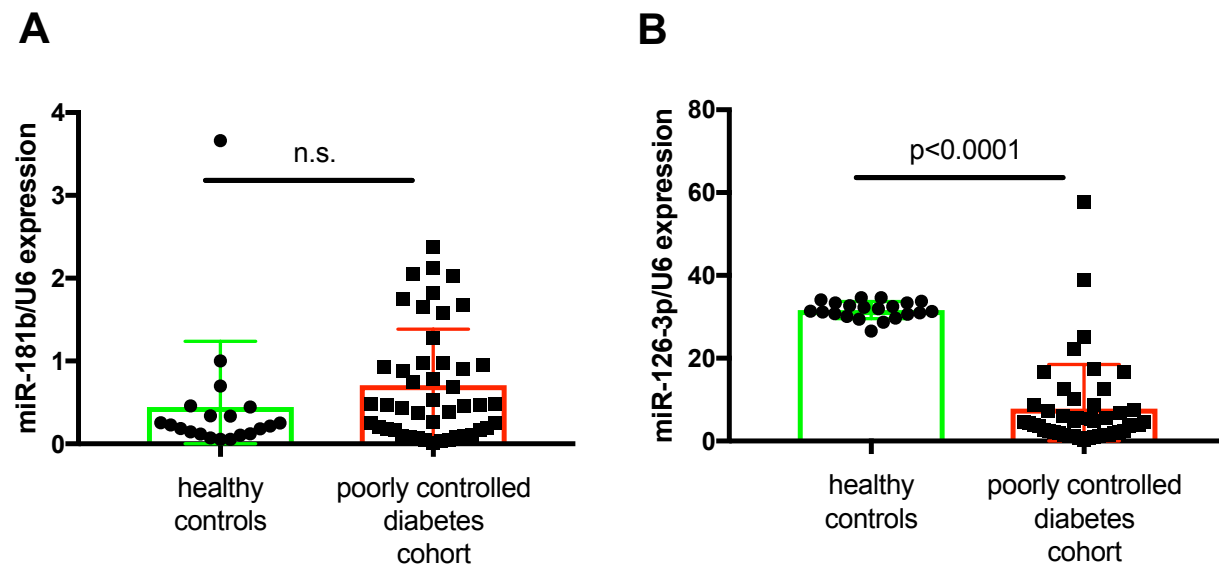

**Figure S5**

Supplement: Supplementary file 6 — Additional file 6: Figure S5. Healthy controls have higher miR-126 but not miR-181b expression than patients with type 2 diabetes. Expression of miR-181b (A) or miR-126 (B) in heathy controls and the cohort with poorly controlled type 2 diabetes. n = 20 for controls, n = 46 for poorly controlled diabetes, p-values by Mann Whitney test. [file 12933_2020_993_MOESM6_ESM.pdf]
